# An Allosteric Potentiator of the Dopamine D1 Receptor Increases Locomotor Activity in Human D1 Knock-In Mice without Causing Stereotypy or Tachyphylaxis[Fn FN3]

**DOI:** 10.1124/jpet.116.236372

**Published:** 2017-01

**Authors:** Kjell A. Svensson, Beverly A. Heinz, John M. Schaus, James P. Beck, Junliang Hao, Joseph H. Krushinski, Matthew R. Reinhard, Michael P. Cohen, Sarah L. Hellman, Brian G. Getman, Xushan Wang, Michelle M. Menezes, Deanna L. Maren, Julie F. Falcone, Wesley H. Anderson, Rebecca A. Wright, S. Michelle Morin, Kelly L. Knopp, Benjamin L. Adams, Borys Rogovoy, Ilya Okun, Todd M. Suter, Michael A. Statnick, Donald R. Gehlert, David L. Nelson, Virginia L. Lucaites, Renee Emkey, Neil W. DeLapp, Todd R. Wiernicki, Jeffrey W. Cramer, Charles R. Yang, Robert F. Bruns

**Affiliations:** Lilly Research Laboratories, Eli Lilly & Co., Indianapolis, Indiana (K.A.S., B.A.H., J.M.S., J.P.B., J.H., J.H.K., M.R.R., M.P.C., S.L.H., B.G.G., X.W., M.M.M., D.L.M., J.F.F., W.H.A., R.A.W., S.M.M., K.L.K., B.L.A., T.M.S., M.A.S., D.R.G., D.L.N., V.L.L., R.E., N.W.D., T.R.W., J.W.C., C.R.Y., R.F.B.); Chemical Diversity, Inc., San Diego, California (B.R., I.O.)

## Abstract

Allosteric potentiators amplify the sensitivity of physiologic control circuits, a mode of action that could provide therapeutic advantages. This hypothesis was tested with the dopamine D1 receptor potentiator DETQ [2-(2,6-dichlorophenyl)-1-((1*S*,3*R*)-3-(hydroxymethyl)-5-(2-hydroxypropan-2-yl)-1-methyl-3,4-dihydroisoquinolin-2(1*H*)-yl)ethan-1-one]. In human embryonic kidney 293 (HEK293) cells expressing the human D1 receptor, DETQ induced a 21-fold leftward shift in the cAMP response to dopamine, with a *K*_b_ of 26 nM. The maximum response to DETQ alone was ∼12% of the maximum response to dopamine, suggesting weak allosteric agonist activity. DETQ was ∼30-fold less potent at rat and mouse D1 receptors and was inactive at the human D5 receptor. To enable studies in rodents, an hD1 knock-in mouse was generated. DETQ (3–20 mg/kg orally) caused a robust (∼10-fold) increase in locomotor activity (LMA) in habituated hD1 mice but was inactive in wild-type mice. The LMA response to DETQ was blocked by the D1 antagonist SCH39166 and was dependent on endogenous dopamine. LMA reached a plateau at higher doses (30–240 mg/kg) even though free brain levels of DETQ continued to increase over the entire dose range. In contrast, the D1 agonists SKF 82958, A-77636, and dihydrexidine showed bell-shaped dose-response curves with a profound reduction in LMA at higher doses; video-tracking confirmed that the reduction in LMA caused by SKF 82958 was due to competing stereotyped behaviors. When dosed daily for 4 days, DETQ continued to elicit an increase in LMA, whereas the D1 agonist A-77636 showed complete tachyphylaxis by day 2. These results confirm that allosteric potentiators may have advantages compared with direct-acting agonists.

## Introduction

Allosteric receptor potentiators increase the affinity and/or efficacy of agonists by binding to a site separate from the orthosteric agonist binding site. Because the response to the potentiator is dependent on the presence of agonist, a potentiator should amplify endogenous control circuits, acting when and where the endogenous agonist is released. (We use the term “potentiator” rather than the more general “positive allosteric modulator” to emphasize that the present study focuses on the consequences of amplifying the response to endogenous agonist.) In contrast, an exogenously administered agonist will activate all the receptors to which it has access for as long as it is present. Given their more physiologic mode of action, allosteric potentiators may have advantages over direct-acting agonists in terms of margin of safety and side-effect profile (Bruns and Fergus, 1990; Epping-Jordan et al., 2007).

It is not difficult to envision other advantages of potentiators. For instance, potentiators may be self-limiting in their actions because they depend on the release of endogenous agonist, which can be down-regulated by the organism in the event of overstimulation. In addition, because the effect of a potentiator will only occur during intermittent release of endogenous agonist, there may be a reduced potential for desensitization. However, there are only a few cases in which these predicted advantages have been documented in vivo, a prime example being the improved safety of benzodiazepines (GABA_A_ receptor potentiators) compared with the older barbiturates (GABA_A_ receptor full allosteric agonists) (Harvey, 1980). Despite the proliferation of allosteric modulators of G-protein-coupled receptors in recent years (May and Christopoulos, 2003; Raddatz et al., 2007), we have been unable to identify examples in which the predicted advantages of G-protein-coupled receptor potentiators have been verified experimentally by side-to-side comparison with direct-acting agonists.

The present report describes initial in vitro and in vivo profiling of DETQ [2-(2,6-dichlorophenyl)-1-((1*S*,3*R*)-3-(hydroxymethyl)-5-(2-hydroxypropan-2-yl)-1-methyl-3,4-dihydroisoquinolin-2(1*H*)-yl)ethan-1-one], a novel allosteric potentiator of the dopamine D1 receptor. DETQ causes a roughly 10-fold increase in locomotor activity (LMA) in transgenic mice expressing the human D1 receptor (hD1 mice) but is inactive in wild-type mice, which have a ∼30-fold lower affinity for DETQ. The response depends on endogenous dopamine tone because it is blocked by a selective D1 antagonist and is not seen in animals in which dopamine levels have been depleted by pretreatment with reserpine.

Interestingly, although D1 agonists show bell-shaped dose–response curves due to competing stereotypical behavior at higher doses, DETQ maintains a plateau in its dose–response curve even though free brain concentrations continue to rise, indicating that the response is self-limiting. In addition, unlike some D1 agonists, DETQ does not show rapid tolerance development on repeated dosing. These results provide experimental confirmation that allosteric potentiators may have clinically relevant advantages compared with orthosteric agonists. The use of DETQ to explore potential therapeutic applications for D1 potentiators will be described in a second paper in this series (Bruns et al., submitted for publication).

## Materials and Methods

### 

#### Materials.

DETQ (referred to as example 3) was synthesized as described elsewhere (Beadle et al., 2014). The acronym DETQ is derived from key chemical groups in the structure: *D*ichlorophenylacetyl 5-(1-hydroxy-1-methyl*E*thyl) *T*etrahydroiso*Q*uinoline. Dopamine agonists and antagonists were purchased from Sigma-Aldrich (St. Louis, MO) except for A-77636 [(1*R*)-3-(1-adamantyl)-1-(aminomethyl)-3,4-dihydro-1*H*-isochromene-5,6-diol;hydrochloride], which was purchased from Tocris (Bristol, United Kingdom). ^3^H-SCH23390 [(6*aS*,13*bR*)-11-chloro-7-methyl-5,6,6*a*,8,9,13*b*-hexahydronaphtho[1,2-*a*][3]benzazepin-12-ol] was obtained from PerkinElmer (Waltham, MA) (NET930; specific activity = 84.3 Ci/mM).

#### Human D1 Cell Line (HEK293).

A human embryonic kidney cell line (HEK293) stably expressing the human D1 receptor was generated via retroviral gene transduction using the pBABE-bleo vector and the Phoenix retroviral system. The cells were grown in Dulbecco’s modified Eagle’s medium/Ham’s F-12 medium (DMEM/F-12) (human D1; GIBCO, Waltham, MA) or DMEM (rat D1; GIBCO) supplemented with 10% fetal bovine serum (FBS), 20 mM HEPES, 2 mM glutamate, and 150 *µ*g/ml zeocin at 37°C in 5% CO_2_.

#### Measurement of cAMP Response in hD1 HEK293 Cells.

At approximately 80% confluency, the cells were harvested using 0.25% trypsin/EDTA, suspended in FBS plus 8% dimethylsulfoxide (DMSO), and stored in liquid nitrogen. On the day of the assay, cells were thawed and resuspended in STIM buffer (Hanks Balanced Salt Solution supplemented with 0.1% bovine serum albumin, 20 mM HEPES, 200 *µ*M IBMX, and 100 *µ*M ascorbic acid). For the potentiator-mode assay, DETQ was serially diluted (1:3) in DMSO and then further diluted 1:40 into STIM buffer lacking dopamine (agonist mode) or containing 2X an EC_20_ (24 nM, or 12 nM final) concentration of dopamine (potentiator mode). (*Note:* In this dilution scheme, the test compound does not come in contact with water until it is diluted into 2X the final concentration. We adopted this scheme because we observed precipitation with compounds from the acyl-tetrahydroisoquinoline series when they were serially diluted in aqueous buffer.). A 25-*µ*l volume of compounds stock solution was mixed with 25 *µ*l of cell suspension (1250 cells) and dispensed into each well of 96-well, half-area plates; the final DMSO concentration was 1.25%. The plates were incubated at room temperature for a total reaction time of 60 minutes.

Cyclic AMP production was quantified using homogeneous time-resolved fluorescence detection (Cisbio, Bedford, MA) according to the vendor’s instructions: lysis buffer containing anti-cAMP cryptate (25 *µ*l) and D2-conjugate (25 *µ*l) was added to the wells, the plates were incubated for an additional 60–90 minutes, and time-resolved fluorescence was detected using an EnVision plate reader (PerkinElmer).

Fluorescence data were converted to cAMP concentrations using a cAMP standard curve and were analyzed using a four-parameter nonlinear logistic equation (ActivityBase v5.3.1.22; IDBS, Guildford, United Kingdom). For potentiator-mode concentration–response curves, the results were expressed as the percentage of the window between an EC_20_ concentration of dopamine alone and the maximum response to dopamine (defined by 5 *μ*M final concentration).

Alpha factor (maximum leftward shift) and *K*_b_ were calculated by fitting the allosteric Schild ratio EC_50_/EC_50_ʹ to the equation *y* = (*K*_b_ + *αx*) / (*K*_b_ + *x*), where *y* is the Schild ratio and *x* is the potentiator concentration (Ehlert, 1988). In the Schild plot, the *x* and *y* axes are both logarithmic.

#### Measurement of cAMP Response in rD1 HEK293 Cells.

A stable HEK293 cell line expressing the rat D1 receptor was created as described earlier for the hD1 cell line, and the cAMP assay was performed as for the hD1 line except that the EC_20_ concentration of dopamine was 5 nM (final) and the cell concentration was 2500 per well.

#### Species Comparison in Transiently Expressed D1 Receptors.

The human, rhesus monkey, dog, or mouse D1 receptor was cloned into the Jump-In vector system (Life Technologies, Austin, TX) and transiently transfected into HEK293 cells using Fugene HD (Promega, Madison, WI). After 2 days of growth in DMEM medium containing 10% FBS, 10 mM HEPES, and 100 *µ*g/ml ampicillin at 37°C in 5% CO_2_, the cells were harvested with TrypLE Express (GIBCO), resuspended in FBS containing 8% DMSO, and stored in 1-ml aliquots (10 × 10^6^ cells/ml) at −70°C. The cAMP accumulation was measured as described for hD1 except that 6000 to 10,000 cells per well were used and the EC_20_ concentration of dopamine used was 0.7 nM (final).

#### Measurement of cAMP Response in hD5 CHO Cells.

A Chinese hamster ovary (CHO) cell line expressing the human D5 receptor was purchased from DiscoveRx (Fremont, CA) (product no. 92-1043). The hD5 assay was performed in the same way as the hD1 assay except that the EC_20_ concentration of dopamine was 2 nM.

#### D2, *β*1, *β*2, *β*3, and 5HT6 cAMP Assays.

The D2, *β*1, *β*2, *β*3, and 5HT6 cAMP assays were performed at DiscoveRx using the protocols described on their website (www.discoverx.com).

#### ^3^H-SCH23390 Binding to Human D1 HEK293 Cell Membranes.

Binding studies were performed using crude membrane homogenates from HEK293 cells stably expressing human D1 receptors. All test solutions were prepared in assay buffer (50 mM Tris-HCl, pH 7.4; 120 mM NaCl, and 1 mM EDTA). Concentration–response curves for dopamine (alone and with three DETQ concentrations) as well as a curve for DETQ alone were preincubated with human D1/HEK293 receptor membranes for 45 minutes at 25°C. Subsequently, ^3^H-SCH23390 was added to a final concentration of 0.6 nM and a final assay volume of 500 *µ*l, with a final DMSO concentration of 1%. Nonspecific and total binding were determined in the presence of 10 *µ*M (+)-butaclamol or assay buffer, respectively.

The reaction was incubated for 75 minutes at 25°C, then bound radioligand was separated from free and collected on a glass fiber filter (GF/C) (PerkinElmer) presoaked in 0.3% polyethyleneimine (Sigma-Aldrich) using a TomTec cell harvester machine. The filter was embedded with Meltilex A solid scintillant (PerkinElmer) and counted on a Wallac Tri-Lux Microbeta (Perkin-Elmer).

Specific binding was calculated by subtracting nonspecific binding from total binding. Specific binding in the presence of test compounds was expressed as a percentage of control specific binding. EC_50_ values for dopamine alone and in the presence of DETQ were calculated using GraphPad software (La Jolla, CA).

#### Generation of Humanized D1 Mice by Gene Replacement.

The targeting vector was based on an approximately 11-kilobase (kb) genomic fragment of the mouse Drd1a gene locus retrieved from the C57Bl/6J RP23 BAC library. A codon-optimized human DRD1 cDNA was synthesized and used to replace the entire mouse Drd1a open reading frame in exon 2. Exon 2 itself was flanked by loxP sites to allow for the generation of a null allele after Cre-mediated recombination [this option was not used in the current studies]. Immediately downstream of the 5′ loxP site in intron 2, an Frt-flanked neomycin resistance cassette was inserted. The 3-kb upstream region of the 5′ loxP site served as a short arm of homology, while approximately 4-kb downstream of the 3′ loxP site constituted the long homology arm. A thymidine kinase cassette was inserted at the far 3′ end to allow for negative selection against random integration.

The quality-tested C57BL/6NTac embryonic stem (ES) cell line was grown on a mitotically inactivated feeder layer composed of mouse embryonic fibroblasts in DMEM High Glucose medium containing 20% FBS (PAN Biotech GmbH) and 1200 U/ml leukemia inhibitory factor (ESG 1107; Millipore, Billerica, MA). We electroporated (Gene Pulser; Bio-Rad Laboratories, Hercules, CA) 1 × 10^7^ cells and 30 *µ*g of linearized DNA targeting vector at 240 V and 500 *µ*F. Positive selection with G418 (200 *µ*g/ml) started on day 2 after electroporation. Selection with ganciclovir (2 mM) started on day 5 after transfection. Resistant ES cell colonies with a distinct morphology were isolated on day 8 after transfection and expanded in 96-well plates. Correctly recombined ES cell clones were identified by Southern blot analysis using several restrictions and external and internal probes and were frozen in liquid nitrogen.

The animal study protocol was approved according to the German Animal Welfare Act (§ 8 (1) TierSchG) by the local authority. The mice were kept in the animal facility at Taconic Biosciences GmbH (Köln, Germany) in microisolator cages (Sealsave; Tecniplast, Buguggiate, Italy). Food and water were available ad libitum. Light was maintained on a 12-hour light/dark cycle, with the light phase starting at 06:00 hours. Temperature and relative humidity were maintained between 21° and 23°C and 45% and 65%.

After administration of hormones, superovulated BALB/c females were mated with BALB/c males. Blastocysts were isolated from the uterus on postcoital day 3.5. For microinjection, blastocysts were placed in a drop of DMEM with 15% fetal calf serum under mineral oil. A flat-tip, piezo-actuated microinjection pipette with an internal diameter of 12–15 *µ*m was used to inject 10–15 targeted C57BL/6NTac ES cells into each blastocyst. After recovery, eight injected blastocysts were transferred to each uterine horn in 2.5-day postcoitus, pseudopregnant NMRI females. Chimerism in the litters (G0) was identified by coat color contribution of ES cells to the BALB/c host (black/white). Highly chimeric male mice were bred to C57BL/6-Tg(CAG-Flpe)2Arte females expressing the Flp recombinase gene. This allowed detection of germline transmission by the presence of black strain C57BL/6 offspring (G1) and generation of the humanized mice by Flp-mediated removal of the neomycin-resistance selection marker in one breeding step. The strain was maintained in the heterozygote state for several generations, with genotyping by polymerase chain reaction (PCR) as described below, after which male and female progeny containing the human D1 receptor were mated, and homozygotes were identified by PCR. Behavior and reproduction of the homozygotes was normal, and the colony was maintained in the homozygote state for succeeding generations.

For genotyping, genomic DNA was extracted from 1- to 2-mm-long tail tips using the NucleoSpin Tissue kit (Macherey-Nagel, Duren, Germany). Genomic DNA (2 *μ*l) was analyzed by PCR in a final volume of 50 *μ*l in the presence of 2.0 mM MgCl_2_ and 200 *μ*M dNTPs, at 100 nM of each primer, and 2 U of Taq DNA polymerase (Invitrogen/Life Technologies, Carlsbad, CA) with primers 3523_51: GGCACAATCCCTATTTAGAGACC and 3524_55: TTTGTTTCAGGCACTCGTGC detecting the presence of the constitutive knock-in allele (259 base pairs). The presence of the wild-type allele in heterozygous mice can be detected with primers 3523_51: GGCACAATCCCTATTTAGAGACC and 3523_52: TGTCTCAGCCTGGTGAGTGC (134 base pairs).

After a denaturing step at 95°C for 5 minutes, 35 cycles of PCR were performed, each consisting of a denaturing step at 95°C for 30 seconds, followed by an annealing phase at 60°C for 30 seconds and an elongation step at 72°C for 1 minute. PCR was finished by a 10-minute extension step at 72°C. Amplified products were analyzed using a LabChip GX device (Caliper Life Sciences/PerkinElmer).

#### Animals, Housing and Care.

Male or female hD1 knock-in mice were bred at Taconic Biosciences (Hudson, NY) and shipped to Eli Lilly in Indianapolis. Animals were roughly 8–12 weeks of age at the time of the study. Age-matched wild-type C57Bl/6 NTac mice were also purchased from Taconic Biosciences. Genotyping was routinely performed by the breeder. All animals were housed in groups of eight mice per cage (shoebox size, 45 × 25 × 20 cm with wood chip bedding) on a 12-hour light/dark cycle with free access to standard animal laboratory food and water. All experiments were performed during the light cycle of the day (6 AM–6 PM). Except when otherwise indicated, the mice were dosed by mouth with an oral gavage using a dose volume of 10 ml/kg. All experimental procedures were preapproved by the local animal care and use ethics committee according to National Institutes of Health guidelines and the Declaration of Helsinki. The Eli Lilly and Company’s animal care and use program is fully accredited by the Association for Assessment and Accreditation of Laboratory Animal Care International.

#### ^3^H-SCH23390 Autoradiography in Wild-Type and hD1 Mice.

Autoradiography was performed essentially as described elsewhere (Camps et al., 1990). Male C57Bl6 and hD1 mice (*n* = 6, Taconic, 8–10 weeks old) were decapitated. The brains were removed, frozen in isopentane on dry ice, and stored at −80°C until sectioned. Frozen sections (20 *µ*m) were cut and thaw-mounted on Fisherbrand Superfrost/Plus microscope slides (Thermo Fisher Scientific, Waltham, MA). Sections were allowed to dry at room temperature then stored frozen at −80°C until used for autoradiography. After 15 minutes of preincubation, binding was conducted in assay buffer (50 mM Tris-HCl, pH 7.4, 120 mM NaCl, 1 mM MgCl_2_, 5 mM KCl, 2 mM CaCl_2_) containing various concentrations of ^3^H-SCH23390 [8-chloro-3-methyl-5-phenyl-1,2,4,5-tetrahydro-3-benzazepin-7-ol] (0.05–10 nM; PerkinElmer). Nonspecific binding was determined by the addition of 10 *µ*M (+)-butaclamol. After 60 minutes of incubation at 22°C, the sections were washed in cold assay buffer (2 × 5 minutes), briefly dipped in water, and air dried. The slides were exposed to Fuji BAS-TR2025 phosphor-imaging plates and digitized with a BAS5000 scanner (FujiFilm, Tokyo, Japan). ^14^C standards were used for image quantitation (American Radiolabeled Chemicals, St. Louis, MO). Quantitative autoradiogram analysis was performed using a computer-assisted image analyzer (Imaging Research, St. Catherines, Ontario, Canada).

#### Locomotor Activity Measurement in Habituated Mice.

Male hD1 mice, 8–10 weeks old (weighing 18 to 25 g at the time of the experiment), were used. Monitoring of mouse LMA took place in transparent plastic shoebox cages having dimensions of 45 × 25 × 20 cm, with a 1-cm depth of wood chips for absorbent bedding, and covered with a ventilated filtered plastic cage top.

Cages were placed in a rectangular frame containing a grid of 12 photocell beams in an 8 × 4 configuration (Kinder Scientific, Poway, CA) that were positioned 2.5 cm from the floor of the cage for the detection of body movements (ambulations), which were recorded by computer for analysis. Mice were placed inside individual LMA boxes for 1 hour during an acclimation period. Mice were then dosed with test compounds (oral or subcutaneous) and were replaced inside individual LMA boxes for the start of LMA. Photocell activity (total ambulations) was collected for 60 minutes using software from Hamilton Kinder (Poway, CA). DETQ was dissolved in 20% 2-hydroxypropyl-*β*-cyclodextrin, and D1 agonists SKF 82958 [9-chloro-5-phenyl-3-prop-2-enyl-1,2,4,5-tetrahydro-3-benzazepine-7,8-diol] and A-77636 and D1 antagonist SCH39166 were dissolved in 0.9% dihydrexidine saline.

Calculation of data based on total ambulations was done using Excel (Microsoft, Redmond, WA). Graphing and statistical analysis of data were done using one-way analysis of variance followed by Dunnett’s multiple comparison test using GraphPad Prism.

#### Video-Tracking Assessment of Horizontal Motion, Rearing, and Grooming in hD1 Mice.

The apparatus for the video-tracking assessment experiment included 12 custom-built video-tracking observation chambers and the HomeCageScan (CleverSys, Reston, VA) software system. The chambers, measuring (in inches) 8 width × 10 length × 8 height, consisted of two end walls, a back wall, a top, and a floor constructed of ¼-in. translucent white acrylic, and one side (front) constructed of clear acrylic. LED strip lighting was placed below the chamber for illumination. A color video camera (640 × 480, 30 frames per second) was placed in front of each chamber to record behavior from a side view. The chambers were located in large sound-attenuating chambers to reduce ambient room noise and isolate lighting. The cameras were connected in groups of four to a quad multiplexer (model NVQC-42N; Nuvico, Englewood, NJ), with each multiplexer connected to its own computer, which then recorded each session to an MPEG file.

At the end of the experiment the files were batch-analyzed by a computer running the CleverSys CageScan video-tracking software. This software can recognize and quantify several different behaviors, including LMA, grooming, rearing, immobility, and feeding behaviors. The current experiment examined behavior in a novel open-field environment rather than home-cage activity, which limited behaviors of interest to LMA, grooming, and rearing.

For HomeCageScan behavioral phenotyping, male D1 knock-in mice (8–12 weeks, 20–25 g body weight) were placed into the observation chambers for a 60-minute habituation session. At the end of the session, the mice were removed from the chambers, dosed with compound (vehicles for DETQ and SKF 82958 were the same as for LMA experiments), and immediately placed back into the chambers for a 60-minute test session. All behavioral activity was monitored and scored by the HomeCageScan system.

#### D1 Receptor Internalization.

These experiments were performed at DiscoveRx. The assay uses a PathHunter cell line in which two inactive complementary portions of *β*-galactosidase are expressed. One *β*-galactosidase fragment is fused to the human D1 receptor, and the other is expressed in the endosome. Enzyme activity is only seen when the two fragments are contiguous.

Cells were seeded in a 20-*µ*l volume into white 384-well plates and preincubated at 37°C for 16 hours. Test compounds were diluted to 5X final concentration in assay buffer (OptiMEM; Invitrogen) plus 0.05% bovine serum albumin) A 5-*µ*l volume of test sample was added, followed by incubation for 30 minutes at room temperature. The final DMSO concentration was 1%.

Subsequently, 5 *µ*l of 6X EC_20_ of dopamine (407 nM final concentration) was added, and the incubation continued for 180 minutes at room temperature. The assay signal was generated by the addition of 15 *µ*l of PathHunter detection reagent cocktail, followed by 60 minutes of incubation at room temperature.

The plates were read with a PerkinElmer Envision instrument fusing chemiluminescent signal detection. The percentage of modulation was calculated using the following formula with RLU (relative light units): % Modulation = 100 × [(Mean RLU of test sample − Mean RLU of EC_20_ dopamine)/(Mean RLU of max dopamine − Mean RLU of EC_20_ dopamine)].

#### Measurement of Free Brain Concentrations of DETQ.

Immediately after the LMA measurements described in Fig. 8, the brains were removed, and brain homogenate concentrations were determined by liquid chromatography with tandem mass spectrometry. The unbound fraction in brain was determined by equilibrium dialysis in a mouse brain homogenate mixture after incubation of the compound for 4.5 hours (Kalvass et al., 2007).

The unbound brain concentrations were calculated from the following equation:


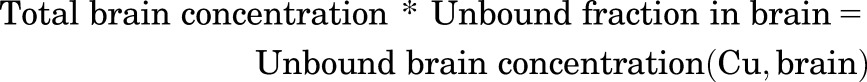


## Results

### 

#### D1 Potentiator Activity of DETQ In Vitro.

**Fig. 1.**
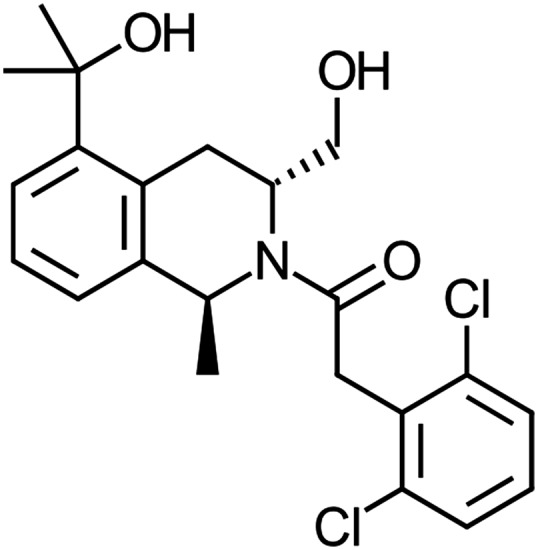
Chemical structure of DETQ.

**Fig. 2.**
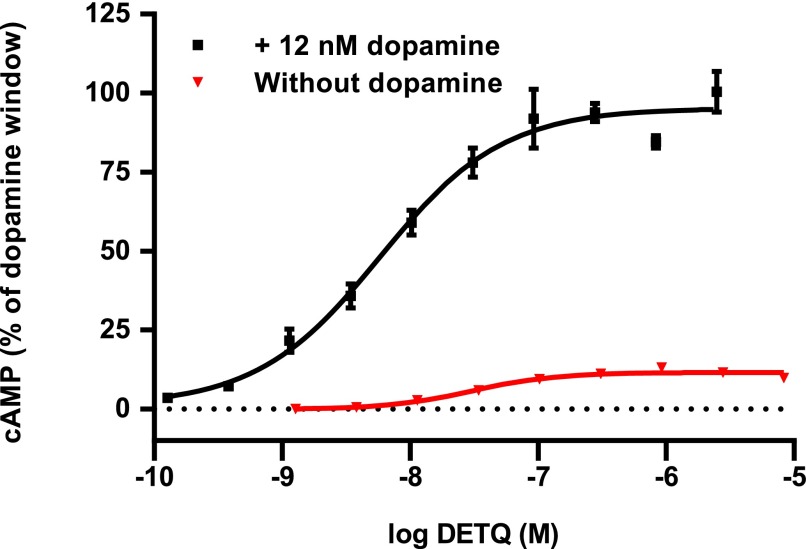
Increase in cAMP by DETQ in the presence and absence of an EC_20_ concentration of dopamine in HEK293 cells expressing the human D1 receptor. Potentiator mode (with 12 nM dopamine): relative EC_50_ 5.83 ± 1.47 nM (S.E.), top 95.2% ± 3.2%, *n* = 5, data from five separate experiments. Agonist mode (without dopamine): relative EC_50_ 30.4 ± 6.9 nM, top 11.6% ± 0.4, *n* = 8, data from a single experiment. In three additional agonist-mode experiments, efficacy of DETQ in the absence of dopamine ranged from 2.7% to 13%, suggesting significant variability in this parameter.

The acyl-tetrahydroisoquinoline DETQ (Fig. 1) originated from chemical modification of a high-throughput screening hit. In HEK293 cells expressing the human D1 receptor, DETQ increased cAMP with an EC_50_ of 5.8 nM when tested in the presence of an EC_20_ concentration of dopamine (Fig. 2). In the absence of dopamine, the response to DETQ was 11.6% of the maximum response to dopamine (Fig. 2), and the EC_50_ in the absence of dopamine was shifted 6-fold to the right, indicating that under these conditions DETQ behaved mostly as a potentiator, with marginal allosteric agonist activity.

**Fig. 3.**
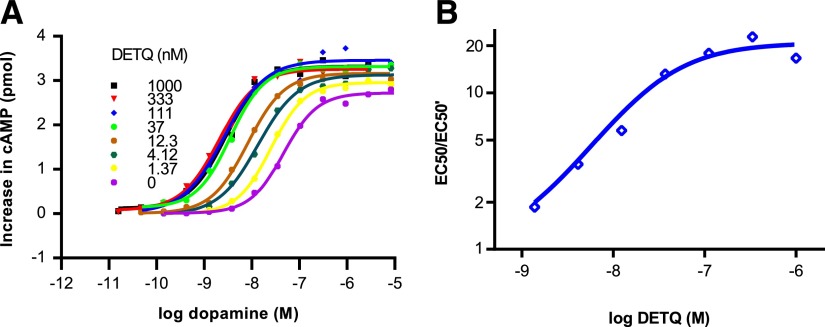
Leftward shift by DETQ of the cAMP response to dopamine in HEK293 cells expressing the human D1 receptor. (A) Dopamine concentration–response curves at different concentration of DETQ. (B) Allosteric Schild plot for DETQ. Best-fit values: *α*-factor 20.8 ± 1.9, *K*_B_ 26.0 ± 10.8 nM.

Relative activity (RA), the efficacy divided by the EC_50_, is a simple measure of potency that is determined by the initial slope of the concentration–response curve (Ehlert, 2008). Comparing RA for potentiator versus agonist mode, DETQ was 43-fold more potent in potentiator mode (Table 1). Dopamine curve-shift experiments (Fig. 3) showed a 20.8-fold leftward shift of the response to dopamine, with a small (22%) increase in the maximum response. The maximum overall sensitization to dopamine, expressed as leftward shift (*α*) times upward shift (*β*), was 20.8 × 1.22 = 25.4 fold.

**TABLE 1:** Relative activity (RA) (Ehlert, 2008) for potentiator mode versus agonist mode for DETQ Relative potency is potentiator mode RA divided by agonist mode RA.

Mode	EC_50_ (nM)	Top (%)	RA	Relative Potency
Potentiator	5.83	95.2	16.33	42.8
Agonist	30.4	11.6	0.38	—

**Fig. 4.**
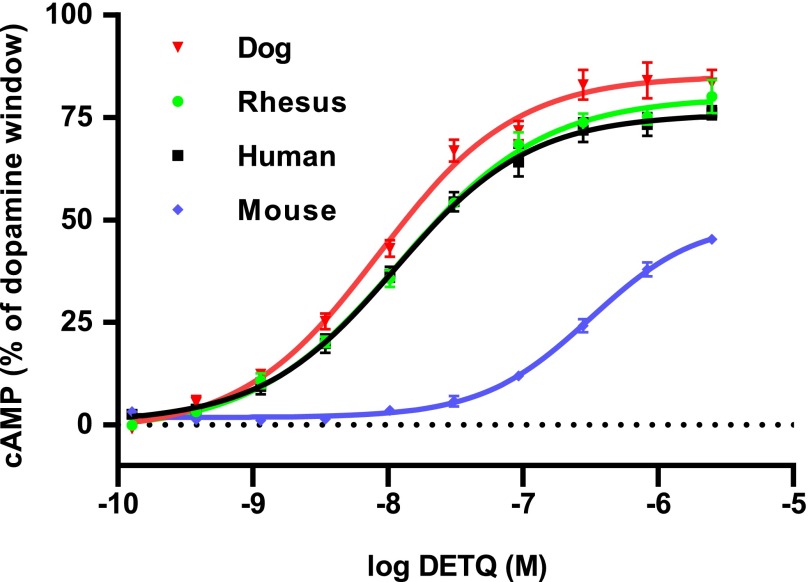
Species differences in affinity for DETQ. Human, dog, rhesus, and mouse D1 receptors were transiently expressed in HEK293 cells. Best-fit parameters from nonlinear curve-fitting are shown in Table 2.

Compared with the human D1 receptor, the functional affinity of DETQ for the rat D1 receptor was 28-fold less (EC_50_ 162 ± 18 nM, *n* = 4). In addition, the maximum response relative to that of dopamine was only 39.9% ± 3.7%, compared with 98.6% ± 4.2% for the human D1. A similar loss of potency was seen for the mouse D1 receptor (Fig. 4 and Table 2), but the rhesus monkey and dog D1 receptors showed nearly identical potency to their human counterpart.

**TABLE 2:** Affinity of DETQ for potentiating the D1-mediated cAMP response in various species Receptors were transiently transfected into HEK293 cells. Values are from nonlinear curve-fitting to a logistic curve. Data points were pooled from experiments carried out on two separate days (n = 8 for all data points).

Species	EC_50_ (nM)	SE	Top (%)	S.E.
Human	11.4	1.6	75.6	1.8
Dog	8.6	1.3	85.2	2.0
Rhesus	12.0	1.6	80.0	1.9
Mouse	312.0	47.4	50.0	2.6

DETQ was inactive at the human D5 receptor in potentiator mode up to 25 *µ*M, and was inactive at the D2, *β*1, *β*2, *β*3, and 5HT6 receptors in potentiator, agonist, and antagonist mode up to 10 *µ*M (Supplemental Data, Supplemental Table 1). DETQ was also inactive at 10 additional targets from the standard Lilly selectivity panel, but showed modest activity at the 5HT2B receptor in antagonist and potentiator mode (respectively 56% inhibition and 48% stimulation at 10 *µ*M, but only 13% and 5% at 1 *µ*M).

**Fig. 5.**
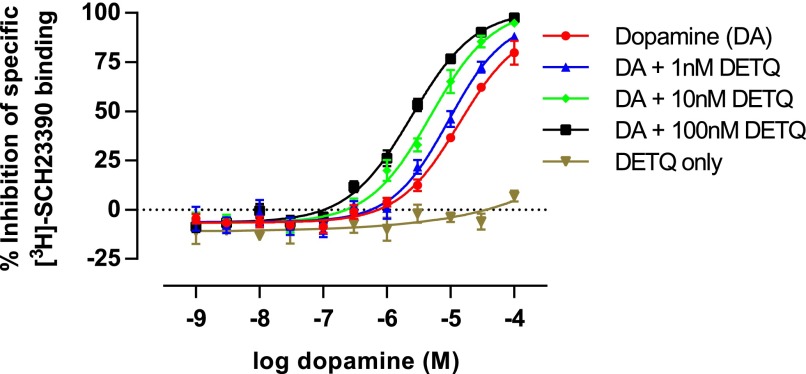
Leftward shift by DETQ of the concentration-inhibition curve to dopamine in ^3^H-SCH23390 binding to membranes from HEK293 cells expressing the human D1 receptor. The values shown are mean ± S.E., *n* = 4. Best-fit IC_50_ values for dopamine were: without DETQ, 13.6 ± 3.4 *µ*M; 1 nM DETQ, 9.4 ± 2.4 *µ*M; 10 nM DETQ, 4.9 ± 0.9 *µ*M; 100 nM DETQ, 2.4 ± 0.3 *µ*M.

DETQ increased the affinity of dopamine to inhibit binding of the D1 receptor antagonist SCH23390 (Fig. 5), indicating that DETQ directly affects the affinity of dopamine for the orthosteric binding site of the D1 receptor.

#### Generation of a Human D1 Receptor Knock-in Mouse.

**Fig. 6.**
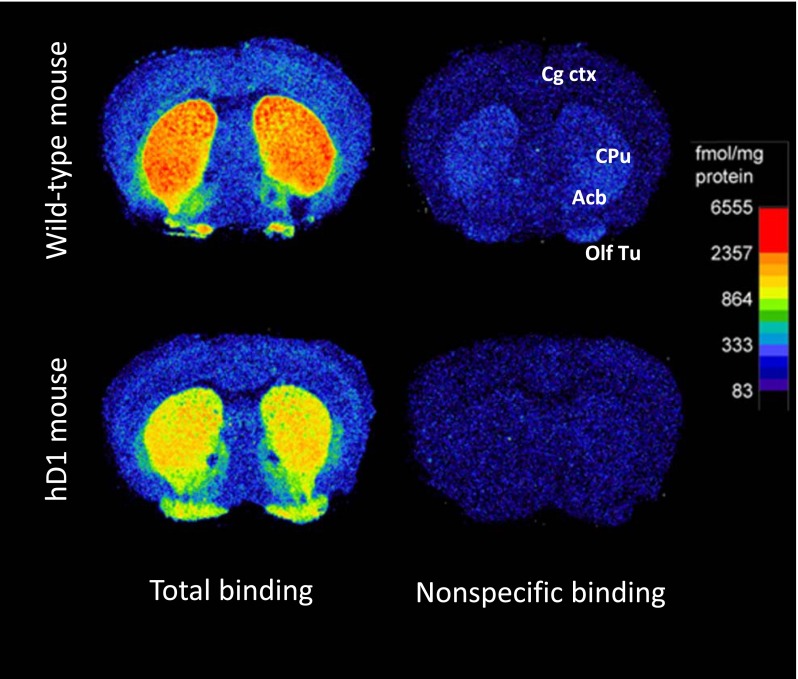
Autoradiograms of ^3^H-SCH23390 binding to coronal sections from brains of wild-type and hD1 mice. High levels of D1 binding sites were seen in the caudate-putamen (CPu), nucleus accumbens (Acb), and olfactory tubercle (Olf Tu) with lower levels of specific binding in the cingular cortex (Cg ctx).

**Fig. 7.**
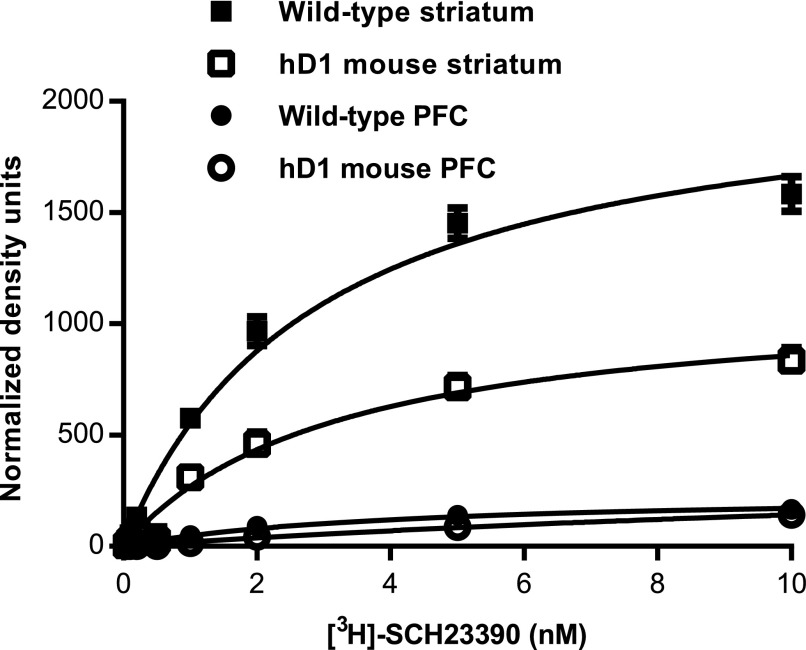
Saturation curves for ^3^H-SCH23390 measured by autoradiography in striatal slices from wild-type and hD1 mice. The values shown are mean ± S.E., *n* = 6. Best-fit values: wild-type, *B*_max_ 2140 ± 130 density units, *K*_d_ 2.89 ± 0.43 nM; hD1 mice, *B*_max_ 1130 ± 90 density units, *K*_d_ 3.22 ± 0.62 nM.

Initial attempts to show in vivo activity of the acyl-tetrahydroisoquinolines in rat and mouse were unsuccessful, as expected given the poor affinity of these compounds for rodent D1 receptors in vitro. To overcome this problem, we commissioned the generation of a transgenic mouse in which the human D1 receptor replaces its murine counterpart. Behavior and breeding of the homozygotes was overtly normal, and the colony was maintained as the homozygote genotype. Distribution of D1 receptor binding in the brain closely resembled distribution in wild-type C57Bl/6 NTac animals (Fig. 6), although D1 receptor density was about 50% lower than in wild-type mice (Fig. 7); a similar difference in receptor density was seen in striatal homogenate binding binding (results not shown).

The IC_50_ values of SCH23390, (+)-butaclamol, and dopamine were similar in wild-type and hD1 mice (Table 3). The concentration–response curve for dopamine in ^3^H-SCH23390 binding was shifted to the left by GTP (data not shown), indicating that coupling to G_s_ was normal. Overall there were no obvious behavioral differences between the two genotypes, and they showed similar behavioral and neurochemical responses to dopamine D1 agonists and antagonists (not shown).

**TABLE 3:** *K*_i_ values for inhibition of 1 nM ^3^H-SCH23390 binding in striatal membranes of wild-type and hD1 mice Results are best-fit parameters from nonlinear curve-fitting to a Langmuir isotherm (n = 3 for all data points).

Compound	Wild-Type Mice		hD1 Mice	
	*K*_i_ (nM)	S.E.	*K*_i_ (nM)	S.E.
SCH23390	0.41	0.04	1.37	0.23
(+)-Butaclamol	2.0	0.4	3.5	0.4
Dopamine	1280.0	420.0	880.0	240.0

#### Increase in LMA Caused by DETQ in hD1 Mice.

**Fig. 8.**
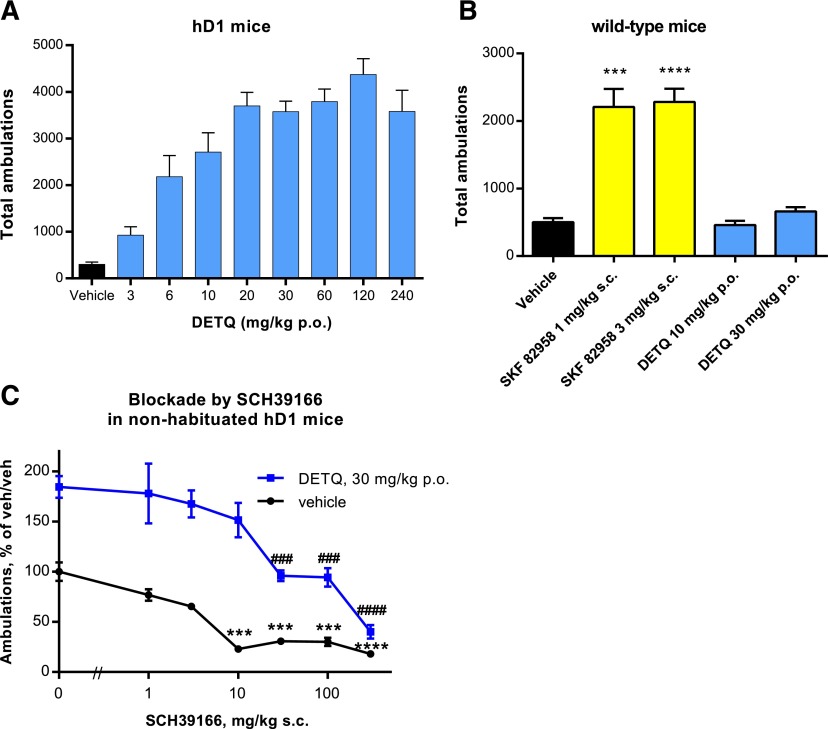
Locomotor response to DETQ in habituated hD1 and wild-type mice. LMA responses in (A) hD1 mice and (B) wild-type C57Bl/6 mice after oral dosing. The values in *A* are mean ± S.E., *n* = 7 to 16. All values for DETQ-treated animals in *A* are significantly different from vehicle (Dunnett’s multiple comparison test on log-transformed data, *P* < 0.0001). The values in *B* are mean ± S.E., *n* = 32 for vehicle and each DETQ dose, and *n* = 8 for each SKF 82958 dose. Groups were compared with vehicle by two-sided *t* test: ***P < 0.001, ****P < 0.0001. (C) Blockade by the D1-selective antagonist SCH39166 of the LMA response to DETQ in nonhabituated hD1 mice. Mice were pretreated for 30 minutes with either vehicle or SCH39166, after which they were treated with either vehicle or DETQ. The LMA was summed over a 1-hour period. The values shown are mean ± S.E., *n* = 20 for vehicle/vehicle, *n* = 15 for vehicle/DETQ, *n* = 5 for all other experimental conditions. The effect of SCH was analyzed by Dunnett’s multiple comparisons test: ***P < 0.001, ****P < 0.0001 for SCH/vehicle versus vehicle/vehicle; ###*P* < 0.001, ####*P* < 0.0001 for SCH/DETQ versus vehicle/DETQ. Additionally, all comparisons between SCH/vehicle and the same dose of SCH with DETQ were statistically significant at the *P* < 0.05 level or better (two-sided *t* test with Welch’s correction for unequal variances).

When mice are introduced to a new environment, they show an initial burst of exploratory behavior. To achieve a lower baseline of activity, all animals were habituated for 1 hour before recording responses to treatment. Transgenic hD1 mice showed a 10-fold dose-related increase in LMA in response to DETQ given orally (Fig. 8). A statistical analysis of responses to various D1 potentiators from the same series as DETQ (W.H. Anderson, unpublished results) showed that a 4-fold increase in LMA consistently showed statistical significance versus vehicle (*P* < 0.05); the dose causing a 4-fold increase in LMA (ED_4X_) was therefore used as an index of potency. The ED_4X_ for DETQ was 7.2 mg/kg. In contrast to the robust LMA response in hD1 mice, in wild-type C57Bl/6 mice 30 mg/kg DETQ increased LMA by only 32%, failing to reach statistical significance (*P* = 0.07) (Fig. 8).

D1 antagonists are known to lower basal LMA (Chipkin et al., 1988), indicating that LMA is dependent on ongoing dopamine release. Given that DETQ showed minimal allosteric agonist activity in vitro, it seemed likely that the LMA response to DETQ was mediated by potentiation of endogenous D1 tone. Confirming this expectation, the LMA response to DETQ was blocked by the D1/D5-selective antagonist SCH39166 (Fig. 8). In nonhabituated mice, SCH39166 lowered basal exploratory activity at 10 *µ*g/kg and lowered DETQ-stimulated activity at 30 *µ*g/kg. The response to DETQ was also absent in hD1 mice whose dopamine levels were profoundly depressed by treatment with reserpine (Bruns et al., submitted for publication).

#### Bell-Shaped LMA Response to D1 Agonists but Not to DETQ.

**Fig. 9.**
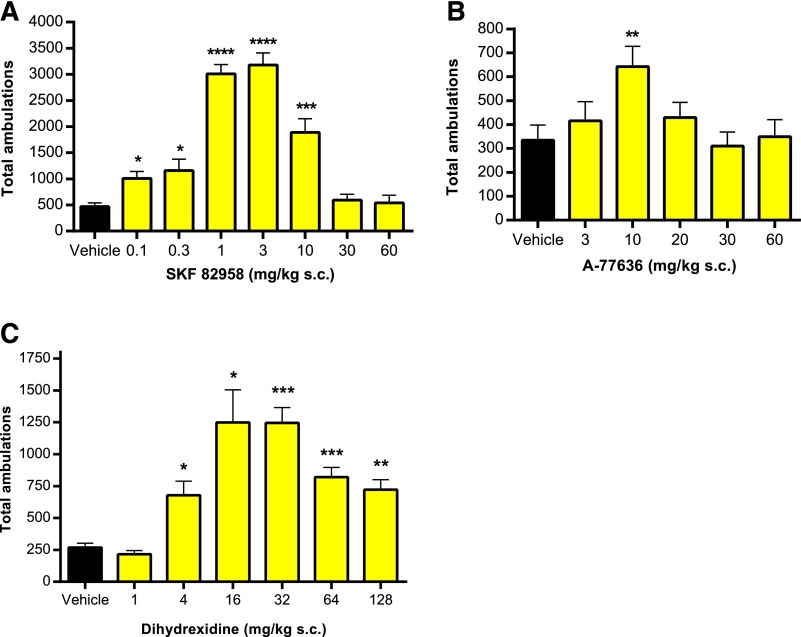
Bell-shaped locomotor responses to the D1 agonists (A) SKF 82958, (B) A-77636, and (C) dihydrexidine. Due to slow onset of activity of A-77636, the values in *B* are from the second hour after dosing. In *A* the *n* values are (from left) 19, 5, 12, 14, 13, 8, 8, 8. In *B*, *n* = 14 for all points; and in *C*, *n* = 9 for vehicle, *n* = 6 for all other points. The values for drug-treated animals were compared with vehicle using a two-sided *t* test: *P < 0.05, **P < 0.01, ***P < 0.001, ****P < 0.0001. In *A* and *C*, Welch’s correction for unequal variances was used.

D1 agonists are known to show bell-shaped (or inverted *U*-shaped) dose–response relationships in various behavioral tests, with activity decreasing at higher doses. The drop-off in LMA at higher doses is typically caused by competition from hyperactivity, with intense stereotyped behaviors such as repetitive grooming (Arnsten et al., 2016) that are incompatible with translational movement. As expected, bell-shaped curves were seen in the hD1 mice with the three D1 agonists, A-77636, SKF 82958, and dihydrexidine (Fig. 9). (*Note:* A fourth D1 agonist, ABT-431 [adrogolide hydrochloride], could not be tested in a wide dose range because it induced seizures in both hD1 and wild-type mice at higher doses; K.A. Svensson, unpublished observation.)

**Fig. 10.**
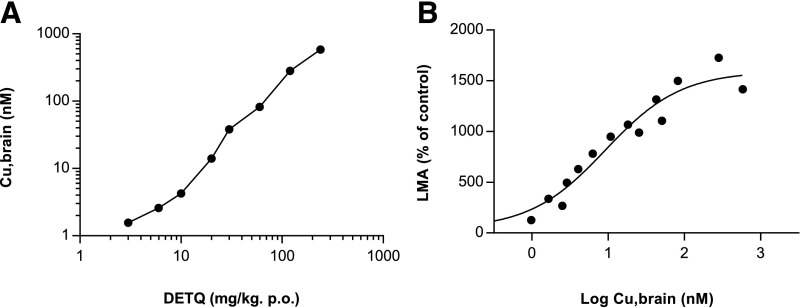
Unbound brain levels of DETQ measured immediately after LMA testing. (A) Dose dependence of calculated unbound brain levels. (B) Relationship between LMA and unbound brain levels. Each point is the mean of brain levels from three animals. The curve is the best fit to a four-parameter model. Best-fit values ± S.E. are: bottom 40% ± 127% of control; top 1,590% ± 130% of control; EC_50_ 9.2 ± 3.1 nM; Hill coefficient 0.88 ± 0.21.

In contrast, the LMA response to DETQ did not show any drop-off at higher doses up to 240 mg/kg (Fig. 8). Free brain levels of DETQ continued to rise at the highest doses (Fig. 10), indicating that the lack of a bell-shaped curve was not due to limited brain exposure. The EC_50_ value of 9.2 nM in LMA in vivo (Fig. 10B) agrees well with the EC_50_ of 5.8 nM for potentiation of dopamine–induced cAMP production in vitro (Fig. 2).

#### Stereotyped Behavior in hD1 Mice Treated with DETQ or D1 Agonists.

**Fig. 11.**
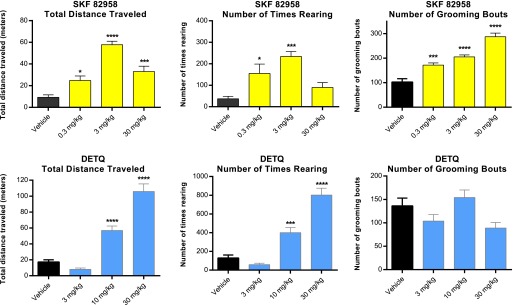
Horizontal motion, rearing, and grooming in response to SKF 82958 and DETQ measured by video-tracking. The values shown are mean ± S.E., *n* = 7–8. Results for drug-treated animals were compared with vehicle using Dunnett’s multiple comparisons test: *P < 0.05, ***P < 0.001, ****P < 0.0001.

Video-tracking was used to compare grooming (an index of stereotyped behavior) with horizontal locomotion and rearing (both indices of exploratory behavior). The D1 agonists SKF 82958 and A-77636 showed an increase in horizontal locomotion and rearing at lower doses; these behaviors were supplanted by grooming at higher doses (Fig. 11). In contrast, DETQ also showed an increase in horizontal locomotion and rearing at lower doses, but this increase was maintained up to the highest dose tested; grooming behavior showed only modest changes that did not reach statistical significance compared with vehicle control (Fig. 11).

#### LMA Responses to DETQ and A-77636 on Repeated Dosing.

**Fig. 12.**
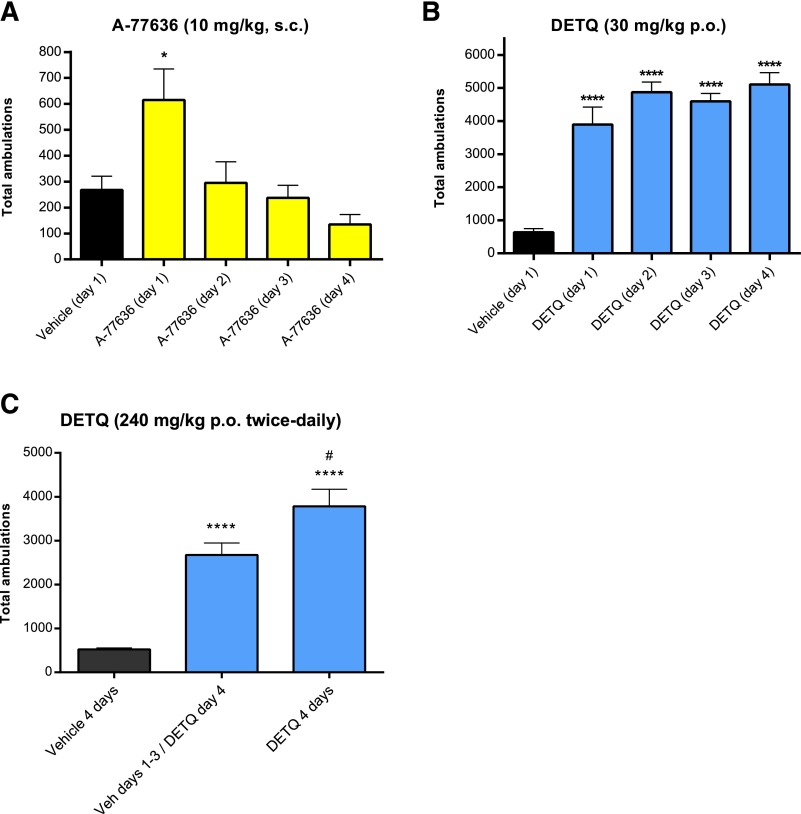
LMA responses to A-77636 and DETQ on repeated dosing. (A, B) Once-daily oral dosing. The LMA response to A-77636 was measured from 60 to 90 minutes because of its slower onset. (C) LMA response to DETQ after twice-daily oral dosing with a high dose of DETQ. The hD1 mice were given 240 mg/kg DETQ (or vehicle) twice daily for 2 days, dosed with 240 mg/kg (or vehicle) on the morning of day 3, then dosed with 240 mg/kg on the morning of day 4, at which time the LMA was measured. The values shown are mean ± S.E., *n* = 7–8. Results for drug-treated animals were compared with vehicle using Dunnett’s multiple comparisons test: (A and B) *P < 0.05, ****P < 0.0001; or a two-sided *t* test: (C) ****P < 0.0001 versus vehicle; #*P* < 0.05 versus animals treated with vehicle on days 1–3 and DETQ on day 4.

Responses to long-acting D1 receptor agonists such as A-77636 (Kebabian et al., 1992) are known to show tachyphylaxis on repeated administration (Gulwadi et al., 2001). In agreement with the literature, the LMA response to A-77636 was completely abolished by day 2 of once-a-day dosing (Fig. 12). In contrast, the LMA response to DETQ was undiminished on day 4 of once-a-day dosing. To check whether the lack of tachyphylaxis was due to the shorter half-life of DETQ, the study was repeated using twice-daily oral dosing of 240 mg/kg. The unbound brain levels of DETQ were 19.8 ± 1.5 nM (*n* = 3) at 6 hours and 30.8 ± 9.4 nM (*n* = 3) at 12 hours Both values are substantially in excess of the in vitro EC_50_ of 5.8 nM for DETQ in the cAMP assay. No tachyphylaxis to DETQ was seen under these conditions; instead, there was a small but statistically significant sensitization. Sensitization on repeated dosing has been previously shown for other dopaminergic agents (Turgeon et al., 1997).

#### D1 Receptor Internalization in Response to DETQ and A-77636.

**Fig. 13.**
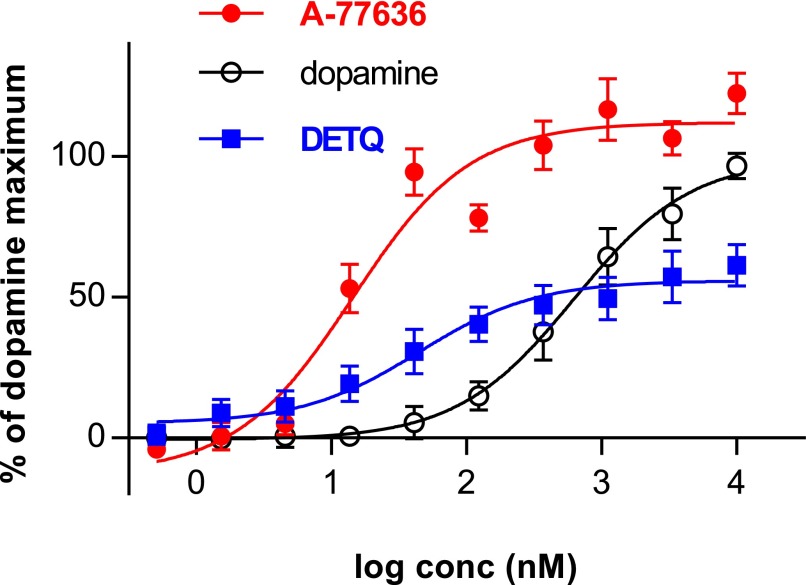
D1 receptor internalization in response to DETQ and A-77636. The values are mean ± S.E., *n* = 6. The best-fit values from a three-parameter model (Langmuir isotherm) are DETQ: bottom 5.0% ± 4.2%, top 55.8% ± 3.7%, EC_50_ 44.6 ± 21.6 nM; A-77636 bottom −12.8% ± 6.7%, top 112.0% ± 3.9%, EC_50_ 14.5 ± 3.7 nM; dopamine bottom −0.3% ± 1.2%, top 99.4 ± 2.7%, EC_50_ 627 ± 69 nM. For DETQ and A-77636, 0% is defined as the response to an EC_20_ of dopamine, and 100% is defined as the maximum dopamine response; for dopamine 0% is the basal internalization, and 100% is the maximum dopamine response.

If DETQ potentiated the cAMP response to dopamine but not the internalization response, this could explain the lack of desensitization seen with DETQ in vivo. To resolve this question, DETQ and A-77636 were tested for effects on D1 receptor internalization. DETQ clearly potentiated the internalization response to dopamine (Fig. 13). (*Note:* Complexation of the D1 receptor with *β*-arrestin is an earlier response along the pathway that leads to internalization. Potencies of dopamine, A-77636, and DETQ in stimulating the *β*-arrestin response were similar to their potencies in stimulating internalization [B.A. Heinz, unpublished observation].) Although DETQ showed a lower maximum internalization rate than A-77636, it should be noted that the maximum response to a potentiator depends on the agonist concentration (for instance, see maximum responses to DETQ at various fixed dopamine concentrations in Fig. 3) and will be higher in the presence of higher agonist concentrations.

A quantitative comparison between DETQ and A-77636 in their effects on the cAMP and internalization pathways was performed using the RA ratio method (Ehlert, 2008). Both compounds were roughly an order of magnitude more potent in stimulating the cAMP pathway than in stimulating the internalization pathway (Table 4). The ratio of pathway preferences (a measure of relative degree of pathway preference between the two compounds) was 2.0 ± 1.4 (Table 4), not significantly different from the ratio of 1.0 expected if the pathway preference was the same for both compounds. These results indicate that DETQ is capable of potentiating the internalization response to dopamine, showing approximately the same degree of pathway preference as the agonist A-77636.

**TABLE 4:** Pathway preference for D1 receptor internalization versus cAMP for DETQ and A-77636 RA is the top divided by the EC_50_. Pathway preference is the RA for cAMP divided by the RA for internalization. Relative preference is the pathway preference of DETQ divided by the pathway preference of A-77636. To ensure comparability, all experiments (including those with A-77636) were performed in the presence of an EC_20_ concentration of dopamine (12 nM for cAMP, 407 nM for internalization). Results for DETQ are from Fig. 2 and Fig. 13. A-77636 internalization results are from Fig. 13. Statistics for A-77636 in the cAMP assay (curve not shown) are: S.E. of the EC_50_ = ±0.86 nM, S.E. of the top = ±17.4% of dopamine max, n = 5. The overall S.E. for relative pathway preference (computed from the S.E.s of the eight components that went into the RA calculations) was ±1.4 (no units).

Compound	Cyclic AMP			Internalization			Pathway Preference	Relative Preference
	EC_50_ (nM)	Top (% of Dopamine)	RA	EC_50_ (nM)	Top (% of Dopamine)	RA	cAMP/Internalization	DETQ/A-77636
DETQ	5.83	95.2	16.3	44.6	55.8	1.25	13.1	1.97
A-77636	2.34	121.0	51.6	14.4	112.0	7.78	6.6	—

## Discussion

This study describes DETQ, a novel, highly selective D1 potentiator. DETQ causes a robust increase in LMA in transgenic mice expressing the human D1 receptor; this response is dependent on endogenous dopaminergic tone. Unlike the response to D1 agonists, the LMA response to DETQ does not drop off at higher doses and does not show tachyphylaxis. These results provide experimental confirmation of some predicted advantages of potentiators over direct-acting agonists.

### 

#### Predicted Advantages of Allosteric Potentiators.

Original research papers (Bruns and Fergus, 1990; Malherbe et al., 2008) and reviews (Christopoulos and Kenakin, 2002; May and Christopoulos, 2003; Epping-Jordan et al., 2007; Arnsten et al., 2016) have emphasized the predicted therapeutic advantages of allosteric potentiators. By amplifying the effectiveness of physiologic circuits, potentiators should give a needed boost to a hypoactive system while allowing the normal feedback loops to remain in control. Because they do not disturb inactive circuits, potentiators should be better tolerated over a wider dose range and be less prone to desensitization compared with direct-acting agonists. However, direct clinical or preclinical evidence confirming these predictions has been difficult to acquire. Using the new tool DETQ, the present study investigates two of these predictions in transgenic mice expressing the human D1 receptor.

The first prediction is the concept that the actions of allosteric potentiators should be self-limiting, because an overstimulated organism can rely on negative feedback loops to reduce neurotransmitter release and de-escalate the system. The D1 system provides an ideal platform for testing this idea because D1 agonists have bell-shaped dose–response curves, stimulating motor activity and cognitive behaviors at moderate doses but inhibiting them at higher doses (Goldman-Rakic et al., 2000; Yang and Chen, 2005). The inhibition of LMA is due to recruitment of stereotyped behaviors such as intense grooming that compete with forward locomotion.

In contrast to the response to D1 agonists, our results show that the LMA response to DETQ reaches a plateau and does not show a declining phase at higher doses. The absence of a declining phase was not due to limited exposure because free brain levels of DETQ continued to rise linearly up to the highest dose tested. The lack of a declining phase implies that allosteric potentiators may have a broader therapeutic window, a potential major advantage over direct agonists. (*Note:* An alternative interpretation of the lack of stereotypy with DETQ could be that the stereotypy is mediated by the D5 receptor, at which DETQ is inactive. We view this as a less likely explanation given the powerful stereotypy seen with D1/D5 agonists and the very low density of D5 receptors in the brain.)

The second prediction is related to the observation that endogenous control systems are often active in bursts (Paladini and Roeper, 2014), turning on when a stimulus is needed and turning off during resting periods. Many systems have mechanisms of desensitization built in to prevent chronic, continuous activation. Because agonists cause continuous activation, they may trigger these desensitization mechanisms. This is the case for long-acting D1 agonists, which show marked tachyphylaxis when dosed for multiple days (Gulwadi et al., 2001). In contrast, a D1 potentiator would be predicted to show less desensitization on chronic exposure because it would only have an effect during those transient periods when dopamine tone was active. Such was the case in our studies. The agonist A-77636 showed a complete loss of responsiveness by day 2 of daily dosing, while DETQ still showed full responsiveness at day 4.

The potential therapeutic advantages described here apply to allosteric potentiators and should be less applicable (or completely inapplicable) to allosteric agonists, defined as allosteric modulators that cause a full response in the absence of agonist. A pertinent clinical example is seen with positive allosteric modulators of the GABA_A_ receptor. Barbiturates are full allosteric agonists at the GABA_A_ receptor (Rho et al., 1996) and can be lethal in overdose due to respiratory depression (Harvey, 1980). In contrast, benzodiazepines are nearly pure allosteric potentiators (Barker et al., 1986) and do not cause severe respiratory depression on overdose unless combined with synergists such as alcohol (Harvey, 1980). Because of this advantage, benzodiazepines (along with structurally different agents that act at the same site) have largely replaced barbiturates in clinical practice (Harvey, 1980). With respect to the above issue, it should be noted that DETQ was 43 times more potent as a potentiator than as an allosteric agonist, indicating that under practical conditions it can be regarded as a pure potentiator. In addition, DETQ did not reverse hypokinesia in hD1 mice pretreated with a high dose of the dopamine-depleting agent reserpine (Bruns et al., submitted for publication), indicating that DETQ lacks direct-acting D1 agonist effects in vivo.

#### D1 Potentiators as Pharmacologic Tools.

DETQ is the first example of a new class of agents that selectively potentiate the function of the dopamine D1 receptor in vivo. D1 receptors have a prominent role in cognition, motivation, and motor activity (Sibley, 1999; Goldman-Rakic et al., 2000; Beaulieu and Gainetdinov, 2011). Based on this role, D1 potentiators may be useful for treatment of several central nervous system disorders, including Parkinson’s disease, Alzheimer’s disease, schizophrenia, major depressive disorder, attention deficit hyperactivity disorder, and narcolepsy. In vivo results supporting these indications are described in a separate study (Bruns et al., submitted for publication).

Several features make DETQ a useful tool for studying the D1 receptor. Its >1000-fold selectivity for the D1 receptor over the closely related D5 is particularly striking, given that numerous attempts to design agonists that distinguish between the two receptors have shown limited success (Giorgioni et al., 2008). Similarly high selectivity versus other related receptors such as D2, 5HT6, and the *β*-adrenergic receptors provides hope that DETQ will be a highly specific pharmacologic tool.

Another advantage of DETQ is its ability to achieve high free brain levels after oral dosing, up to ∼600 nM at 240 mg/kg (Fig. 10). This is nearly two orders of magnitude higher than the free brain EC_50_ of 9.2 nM, implying ample headroom for dosing in animal studies. Furthermore, no overt adverse behavioral effects were observed at any of the doses, consistent with the lack of off-target activity seen in vitro.

A potential disadvantage of DETQ is its lower affinity for the mouse and rat D1 receptors, which necessitated the generation of a transgenic hD1 mouse to show in vivo activity. However, our results with the dog (Table 2) and rabbit (unpublished observations) D1 receptors suggest that the lower affinity is limited to rodents. Efficacy in the nonhuman primate has also been confirmed using the spontaneous eye-blink model (Bruns et al., submitted for publication). Interestingly, the lower affinity of the Bristol-Myers Squibb D1 potentiator “Compound B” (Lewis et al., 2015) for the rat receptor was traced to an R130Q mutation that is limited to the rodent clade (http://www.uniprot.org/).

#### Unique Pharmacology of Allosteric Potentiators.

Allosteric potentiators have a unique place as pharmacologic tools (Conn et al., 2009; Canals et al., 2011; Keov et al., 2011). Whereas antagonists work by subtraction and agonists work by addition, potentiators work by multiplication. That is to say, a potentiator increases the sensitivity of its target receptor to its endogenous neurotransmitter or hormone, amplifying the physiologic role of that receptor. In contrast, an agonist will activate its receptor regardless of whether the natural ligand is present, leaving unanswered the question of what is physiologic and what is only pharmacologic. Furthermore, a potentiator provides qualitatively opposite information compared with an antagonist: with an antagonist, one can study a physiologic function by observing its disappearance whereas with a potentiator one can observe an amplification or exaggeration of the function, which can be an advantage if the purpose is to characterize the function in detail. For instance, the ability of DETQ to stimulate locomotor activity in hD1 mice shows that D1 receptors have a tonic, ongoing role in maintaining motor activity. This result was not surprising in light of prior knowledge that selective D1/D5 antagonists suppress basal motor activity (Chipkin et al., 1988). However, the wide dynamic range of the D1 system, illustrated by the ability of DETQ to increase motor activity by more than 10-fold, is something that could not have been deduced from studies of D1 antagonists.

#### Desensitization and Accommodation.

In interpreting these studies, it is important to distinguish between two phenomena, which we will refer to as desensitization and accommodation. We define desensitization as refractoriness to additional input so that an input that normally would produce a response no longer does so. An example would be the tachyphylaxis to D1 agonists upon chronic dosing. In contrast, we define accommodation as the ability to adjust an input to produce the correct magnitude of output. A potential example would be the lack of stereotypy shown by DETQ, in that animals do not show signs of overstimulation even when exposed to extremely high free brain levels of DETQ. Our studies show that potentiators allow accommodation without causing desensitization, a unique profile that should confer therapeutic advantages.

#### Conclusions.

In the present studies, we have used the novel D1 potentiator DETQ to verify two predicted advantages of allosteric potentiators. In contrast to D1 agonists, DETQ increases LMA without causing stereotypy at high doses, confirming that potentiators may have broader therapeutic windows compared with agonists. In addition, unlike D1 agonists, DETQ did not cause tachyphylaxis on repeated dosing, indicating that potentiators may have better ability to maintain efficacy on chronic dosing. We recommend that these advantages of potentiators be explored in clinical studies.

## Abbreviations

A-77636: (1*R*)-3-(1-adamantyl)-1-(aminomethyl)-3,4-dihydro-1*H*-isochromene-5,6-diol,; hydrochloride
ABT-431: adrogolide hydrochloride
DETQ: 2-(2,6-dichlorophenyl)-1-((1*S*,3*R*)-3-(hydroxymethyl)-5-(2-hydroxypropan-2-yl)-1-methyl-3,4-dihydroisoquinolin-2(1*H*)-yl)ethan-1-one
DMEM: Dulbecco’s modified Eagle’s medium
DMSO: dimethylsulfoxide
ES: embryonic stem
FBS: fetal bovine serum
hD1 mouse: human D1 knock-in mouse
HEK293: human embryonic kidney 293
kb: kilobase
LMA: locomotor activity
PCR: polymerase chain reaction
RA: relative activity
RLU: relative light units
SCH23390: 8-chloro-3-methyl-5-phenyl-1,2,4,5-tetrahydro-3-benzazepin-7-ol
SCH39166: (6*aS*,13*bR*)-11-chloro-7-methyl-5,6,6*a*,8,9,13*b*-hexahydronaphtho[1,2-*a*][3]benzazepin-12-ol
SKF 82958: 9-chloro-5-phenyl-3-prop-2-enyl-1,2,4,5-tetrahydro-3-benzazepine-7,8-diol
